# Sensitive light-sheet microscopy in multiwell plates using an AFM cantilever

**DOI:** 10.1364/BOE.9.005863

**Published:** 2018-11-02

**Authors:** Aleks Ponjavic, Yu Ye, Ernest Laue, Steven F. Lee, David Klenerman

**Affiliations:** 1 Department of Chemistry, University of Cambridge, Cambridge, United Kingdom; 2 Department of Biochemistry, University of Cambridge, Cambridge, United Kingdom; 3 Department of Cell Biology, Harvard Medical School, Longwood Avenue, Boston, 02115 MA, USA; 4 ap876@cam.ac.uk; 5 sl591@cam.ac.uk; 6 dk10012@cam.ac.uk

## Abstract

We present a sensitive inverted light sheet microscope, capable of single-molecule fluorescence imaging of cells in 96-well plates. Light sheet microscope designs are often complex and costly, requiring custom-made sample chambers that are incompatible with standard cell culture samples. To overcome this limitation, we have developed single-objective cantilever selective plane illumination microscopy (socSPIM), which introduces a light sheet through the objective lens of an inverted microscope using an AFM tip. We demonstrate the effectiveness of this setup by performing 3D imaging of nuclear pore complexes, as well as live whole-cell 3D imaging of lysosomes and super-resolution imaging of the T-cell membrane. The unique advantage offered by socSPIM is the minimal footprint of the cantilever, which allowed us to perform super-resolution reflected light-sheet microscopy by PAINT in 96-well plates, paving the way for high-throughput studies.

## 1. Introduction

By matching the excitation volume to the axial depth of field of the microscope, light-sheet fluorescence microscopy (LSFM) reduces out-of-focus excitation and photobleaching of molecules outside the imaging plane, which is a problem that is prevalent in many other fluorescence imaging methods [1]. It therefore allows deep intracellular imaging with minimal phototoxicity and photobleaching [2]. LSFM contrasts with the widely used total internal reflection microscopy, where one is limited to imaging within ~200 nm of the water-coverslip interface [3]. This surface limitation can also be overcome using highly inclined and laminated optical sheet (HILO) microscopy [4], but for whole cell imaging the minimal thickness of the illuminated volume with HILO is on the order of 6 μm, whereas LSFM can create a sheet of light with a thickness of about 1 μm [5]. The improved reduction in background excitation with LSFM results in superior contrast for applications such as single-molecule fluorescence imaging [6] and can therefore be used to improve localization-based super-resolution microscopy [7]. Despite all these advantages, the widespread use of LSFM has been somewhat limited by high technical complexity and the specific design requirements imposed on microscopes [8]. High-throughput super-resolution methods in particular [9] would greatly benefit from the superior contrast afforded by LSFM, as it would improve localization precision [10] and enable imaging of larger cellular structures [11]. In this manuscript, we describe an implementation of LSFM that can easily be implemented on commercial inverted microscopes, and which is compatible with imaging cells on coverslips, in petri dishes and in 96-well plates.

A difficulty with implementing LSFM is that the excitation light has to be brought in perpendicular to the detection objective. This has spawned numerous implementations of LSFM [12], which all provide unique solutions to the same problem. Typically, a secondary light-sheet objective lens is positioned perpendicular to the detection objective to create the light sheet [13]. The physical geometry of the two objective lenses and the necessity of positioning them both very close to the sample often limits the numerical aperture (NA) of the light-sheet objective as well as the detection objective, limiting both axial resolution and collection efficiency. Furthermore, normally there is a need to create a flat interface through which the light can enter, which requires the design of complex custom-made sample chambers [13–15]. These constraints can be alleviated by introducing light via the placement of a reflective mirror close to the sample, allowing the detection objective to also be used for excitation. This single-objective selective plane illumination microscopy (soSPIM) [11] design used photolithography fabrication methods to incorporate 45° mirrors. This approach, however, requires bespoke and therefore expensive sample holders [10,16].

An alternative method to implement LSFM, that can be used with standard samples, is to place a reflective atomic force microscopy (AFM) tip near the sample, and introduce light through a water dipping objective above the sample [17,18]. It is also possible to create a two-objective perpendicular geometry in custom-made microscopes by dipping both excitation and detective objectives into the sample solution [19,20]. While, these setups are compatible with standard sample geometries, the two-objective design has a large footprint that is incompatible with inaccessible sample geometries such as 96-well plates. Oblique angle microscopy offers a single-objective solution to the problem by using a highly angled light sheet and tilting the imaging plane using an additional objective [21], which has been applied to high-throughput 3D LSFM imaging in 96-well plates [22]. However, this comes at a cost of being limited to low NA objectives, with thicker sheets (FWHM ∼6 μm) and inefficiencies in light collection that preclude low light applications such as single-molecule imaging. Consequently, there is a need to implement the advantages of soSPIM in a way that is suitable for widespread adoption by the biology community.

In this paper, we present an evolution of soSPIM, which we call single-objective cantilever selective plane illumination microscopy or socSPIM. Our approach introduces a light sheet through the objective lens of an inverted microscope using a standard cantilever AFM mirror [17], used in a non-conventional way, to provide a reflective interface for imaging using high NA objectives on inverted microscopes that are compatible with multiwell plates. We show that this configuration performs similarly to other high NA light sheet methods by imaging the nuclear pore complex (NPC) in fixed cells and comparing the results to the commonly used HILO excitation [4]. The new microscope is then used to undertake live whole-cell 3D imaging of lysosomes in HEK cells at a frame rate of 0.5 Hz. Finally, we present super-resolution LSFM imaging, by points accumulation for imaging in nanoscale topography (PAINT) [23], in a 96-well plate for the first time.

## 2. Materials and methods

### 2.1 Optical setup

**Fig. 1:**
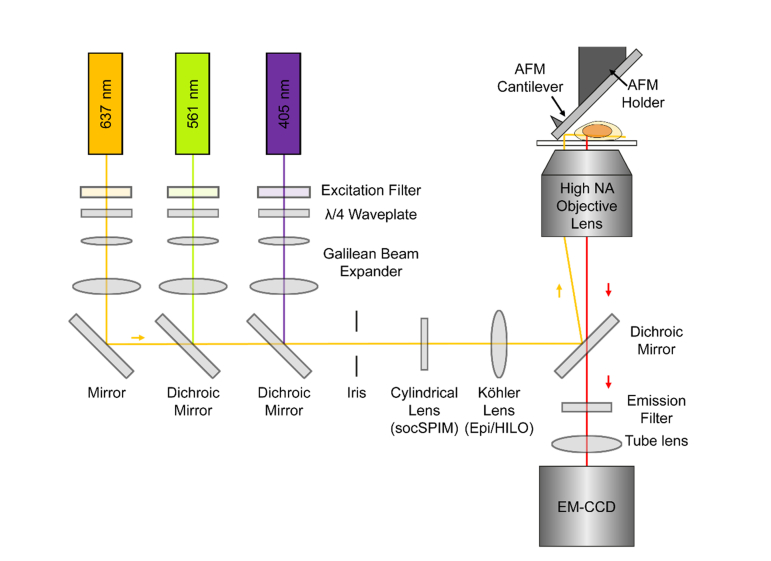
Schematic of socSPIM setup. Three laser beams of different wavelengths are guided into a high NA objective lens via mirrors. The beams are reflected by an AFM cantilever mounted onto a metallic rod holder. In socSPIM mode a cylindrical lens is used to create a light sheet in the image plane. By replacing the cylindrical lens with a spherical lens, the setup can be used in epifluorescence or HILO mode for comparison. The emission is captured by an EM-CCD camera.

The socSPIM light sheet was implemented on a standard inverted microscope (Nikon Eclipse Ti-U) set to an internal magnification of 1.5×. A schematic of the optical setup is shown in Fig. 1

.

405 nm (500 mW OFL510, OdicForce Lasers), 561 nm (50 mW DPL561, Cobolt) and 640 nm (20 mW iFLEX-2000, Qioptic) laser lines, with excitation filters (LL02-561-25 and FF01-640/14-25, Semrock), were expanded and collimated using Galilean beam expanders and then combined using dichroic mirrors. Either a 100× (150× with internal magnification) 1.49 NA oil immersion objective (Apochromat TIRF, MRD01691, Nikon) or a 60× (90× with internal magnification) 1.27 NA water immersion objective (Nikon Plan Apochromat, MRD07650) was used to focus the light onto the sample and to collect the fluorescence emission. An UltraFlat (<0.25 lambda/inch) quad-band dichroic mirror (ZT405/488/561/640rpc-UF3, Chroma) was used in the microscope to separate excitation and emission. The excitation beams have to travel farther than the emission, as they have to be reflected by the AFM mirror. To compensate for this increased distance, each laser line was slightly decollimated using the beam expander to offset the focus of the laser beams from the depth of field of the microscope. This was achieved by imaging the reflection of the beam 25 μm below the coverslip/water interface and tuning the Galilean beam expander such that the beam is in focus. Because a reflection is being imaged, this 25 μm offset corresponds to an increase in the combined focal length of 50 μm. A cylindrical lens was used to focus the light in one axis onto the back focal plane of the objective, resulting in a collimated output such that the final light sheet only focuses in one dimension. The microscope could also be operated in HILO mode by replacing the cylindrical lens with a spherical lens with the same focal length. This lens and the final mirror directing light into the microscope were placed on an x-stage, enabling lateral displacement of the laser beam as is often done for total internal reflection microscopy (TIRFM) and HILO excitation [4]. For HILO excitation, an iris was used to make the excitation footprint ~20 μm in diameter to be slightly larger than a cell for optimal sectioning. The emitted fluorescence was then filtered using emission filters (LL02-561-25, Semrock or #67-038, Edmund Optics) and finally focused using the tube lens onto the EMCCD camera (Evolve 512 Delta, Photometrics), operating in frame transfer, clear pre- sequence with an EM gain of 250. The pixel sizes were 106.7 (150×) and 177.7 (90×) nm, which was confirmed using a 1951 USAF Hi-resolution target (Edmund Optics).

The sample stage was mounted onto an xyz piezo (P-611.3 Nanocube, Physik Instrumente) to enable fast and accurate scanning of samples. A machined brass or aluminium rod with dimensions 50×2.5×2.5 mm was used to provide an inclined surface (43°), onto which an AFM cantilever was attached using cyanoacrylate adhesive (Fig. 4). The commercial AFM cantilever (ContAl-G, BudgetSensors) has a reflective Al coating. These tips are typically not recommended for liquid AFM, due to the propensity of the delamination of the Al coating, but we did not observe any noticeable degradation in the quality of the generated light sheet over the course of hours. The brass rod supporting the cantilever was attached to a manual xyz-stage for positioning of the cantilever mirror and thus the light sheet.

The thickness of the light sheet was determined by imaging a 100 nM solution of Oxazine 725 in PBS, excited using the 637 nm laser. 100 ms exposure images were taken for two different aperture sizes as a means of varying the effective NA and thus the Rayleigh length. The intensity distribution was then analyzed by fitting Gaussian functions as shown in Fig. 4d. The variation in the Gaussian radius as a function of position could then be fitted to determine the Rayleigh length, which was determined to be 4-10 μm in our implementation depending on the optical setup used.

### 2.2 socSPIM alignment

**Fig. 2:**
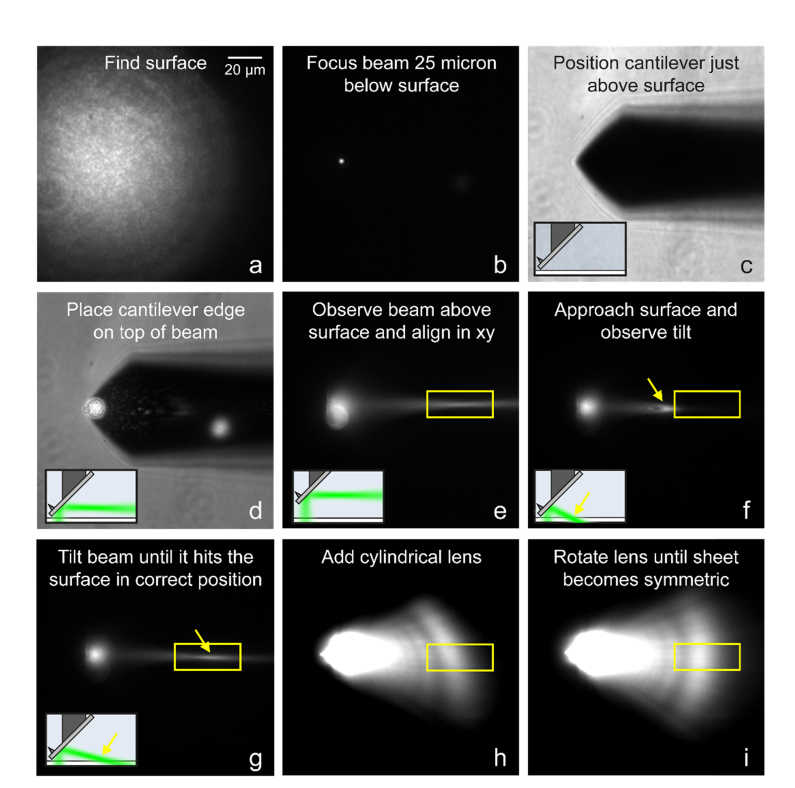
socSPIM alignment. Insets show the position of the cantilever relative to the coverslip and the angle of the light sheet as viewed from the side. a: Epifluorescence image of Oxazine 725 adsorbed to a coverslip. b: Reflection of laser beam at the glass-water interface can be observed, focused 25 μm below the interface in (a). c: The AFM cantilever is positioned just above the coverslip surface. d: The bottom edge of the cantilever is positioned on top of the laser beam. e: The cantilever is lifted from the surface to observe the focal point of the laser beam in solution using fluorescence. f: An example of having too steep of an angle is shown when the cantilever is close to the surface. g: By tilting the angle properly the laser beam hits the surface within its focal depth. h: A cylindrical lens creates a sheet of light in the image plane. i: Rotation of the cylindrical lens properly aligns the sheet.

Alignment has to be carried out whenever a new AFM tip is mounted onto the machined rod. As long as the mounting of the machined rod onto the xyz-stage or piezo is stable, realigning every time the rod is removed for cleaning is typically not necessary. It is also not necessary to realign when moving between wells on a multiwell plate, or when changing petri dishes and coverslips. Our alignment procedure for socSPIM is depicted in Fig. 2

. In this example a 50 μl solution of 100 nM Oxazine 725 in PBS was placed onto a glass coverslip and a 1.27 NA water objective was used for imaging.

Step 1: Focus on the surface by observing the fluorescence of Oxazine 725 adsorbed to the coverslip (Fig. 2(a)). This can be done in any excitation mode but epifluorescence simplifies finding the surface.

Step 2: Lower the laser power using density filters and remove emission filters to observe the laser beam reflected at the coverslip-water interface. Lower the focal plane by about 25 μm either using a piezo or if none is available use the known calibration for the microscope focus knob. Remove the Köhler lens and decollimate the beam using the Galilean beam expander until the beam is in focus (Fig. 2(b)). Position the beam off-center in the direction of the cantilever tip and then align it so that it is straight along the optical axis.

Step 3: Focus on the surface again. Now lower the cantilever towards the surface using the xyz-stage it is mounted on. The cantilever can be observed using white light illumination (Fig. 2(c)). Approach the surface until the cantilever touches the coverslip. This can be observed visually as it will start to move laterally due to bending when it comes into contact. Then retract the cantilever slightly (1-2 μm if you have a piezo), such that it is close to the surface but not in contact.

Step 4: Remove emission filters again and tune the laser power and white light such that both the cantilever and the scattered light of the laser beam can be observed simultaneously (Fig. 2(d)). Now position the tip of the cantilever on top of the beam.

Step 5: Raise the cantilever off the surface and observe the laser beam with fluorescence. The focal point of the beam should be visible (Fig. 2(e)). At this point, the beam might not be straight in the image plane due to how the cantilever is mounted. Use one of the laser mirrors to straighten the beam. If a different focal position is desired the decollimation can now be modified to translate the focal point of the beam to the desired position. Now record the focal point position (yellow box in Fig. 2(e)). Note that if an entire cell is to be imaged, there needs to be sufficient distance for the beam to reach the surface given the small inclination angle, which is why we recommend a distance of at least 30 μm from the tip.

Step 6: Approach the surface again with the cantilever as done in step 3. Now observe with fluorescence where the laser beam illuminates the surface. Because the dye adsorbs on the surface this can be seen as an area of increased intensity (yellow arrow in Fig. 2(f)). If the angle is too steep (Fig. 2(f)), the beam will hit the coverslip before the identified focal point (yellow box in Fig. 2(f)). If the angle is too shallow, no area of increased intensity will be observed.

Step 7: Change the tilt angle using one of the laser beam mirrors until the laser beam hits the surface within the focal point of the beam (Fig. 2(g)).

Step 8: Mount the cylindrical lens to turn the focused laser beam into a sheet (Fig. 2(h)).

Step 9: Finally, rotate the cylindrical lens until the sheet is flat (Fig. 2(i)).

### 2.3 3D imaging of nuclear pore complexes

Prior to imaging, HEK (HEK293A).were grown overnight on plasma-cleaned glass coverslips in OptiMEM without FBS. Cells on the coverslips were carefully washed three times with filtered OptiMEM and subsequently fixed for an hour using PBS containing 4% paraformaldehyde and 0.2% glutaraldehyde. The fixed cells were then permeabilized using 0.1% TX-100 in PBS (10 min) and stained with mAb414 antibody against NUP labelled with Alexa Fluor 647 (BioLegend). The antibody was removed after 1 hr of staining and washed three times in PBS containing 0.1% Tween-20. Filtered PBS was used for final imaging of the fixed cells.

Cells were imaged with the 1.27 NA water immersion objective lens using the 637 nm laser line for excitation at a power density of approximately 0.01 kW/cm^2^, estimated based on the background-subtracted signal when compared with epifluorescence illumination [14]. The epifluorescence power density was determined by measuring the laser power on top of the objective and measuring the footprint of the laser at the coverslip. A low power was chosen to ensure minimal photobleaching of cells to allow a fair comparison between socSPIM and HILO excitation. Cells were identified using white light images and for each cell a z-stack of 60 frames in steps of 200 nm was taken using socSPIM excitation with an exposure time of 33 ms. Subsequently, HILO mode was used to image the same cells and the power for HILO was matched to the background-subtracted signal of NUP when excited by socSPIM.

### 2.4 Live 3D imaging of lysosomes

A plasmid expressing rat Lamp1 carrying a tandem N-terminal fusion to mEos4b (Addgene 57512) was transfected into HEK293A cells and grown in DMEM media for 48 hrs. Prior to imaging, cells were grown overnight on plasma-cleaned glass coverslips in OptiMEM without FBS. Cells on the coverslips were carefully washed with filtered OptiMEM and subsequently mounted for imaging.

Cells were imaged with the 1.27 NA water immersion objective lens using the 561 nm laser line for excitation (at a power density of 0.02 kW/cm^2^). Prior to imaging, a 1 second burst of low power (0.1 mW) 405 nm illumination was used to photoactivate a portion of the mEos4b with epifluorescence excitation. A z-stack consisting of 40 frames at 500 nm steps were taken for each time point, with a timestep of 2 s using an exposure time of 33 ms for each frame. The data presented in Fig. 8 was created by deconvolution using the Richardson-Lucy algorithm with the FIJI plugin DeconvolutionLab2 [24] with a simulated Gaussian PSF based on the light sheet thickness measurement. To account for photobleaching, the mean intensity of each frame was normalized to the first by dividing the intensity in each pixel by a constant value for each.

### 2.5 Super-resolution PAINT imaging of T-cell membrane

Jurkat T cells (Clone E6-1, ATCC TIB-152) were grown in RPMI medium supplemented with 10% FCS, 1% HEPES buffer (1M), 1% sodium pyruvate (100mM), 2% L-glutamine (200mM) and 1% penicillin-streptomycin.

For the HILO-socSPIM comparison 10 μl of 10^6^ Jurkat T cells/ml was added to a coverslip coated with poly-L-lysine (0.01%, Sigma-Aldrich). After 10 minutes, 35 μl of 1 mg/ml bovine serum albumin (BSA) in PBS was added to block the surface. Finally, 5 μl of 200 nM WGA labelled with Alexa Fluor-555 (W32464, ThermoFisher Scientific) in 1 mg/ml BSA in PBS was added to the coverslip. The WGA binds to glycosylated proteins on the cell membrane. 3500 frames at 50 ms exposure and 20 Hz were taken for a single cell using light-sheet excitation with the 561 nm laser set to a power density of 0.02 kW/cm^2^. The same cell was then imaged in the same plane using HILO excitation at a similar power density achieved by approximately matching the intensity of localizations.

For the 96-well plate experiments, 50 μl of 10^6^ Jurkat T cells/ml was deposited into six different wells of a 96-well plate (CELLCOAT 655946, Greiner Bio-One), coated with poly-D-lysine. After allowing the cells to attach for 10 minutes, 45 μl of 1 mg/ml BSA in PBS was added to each well to block the electrostatic poly-D-lysine surface, as well as to limit adhesion of dyes to the AFM cantilever. WGA-555 was dissolved in PBS, containing 1 mg/ml BSA in PBS at six different concentrations, covering more than two orders of magnitude. 5 μl of each stock was added to the six wells. Cells were imaged using the light sheet with the 561 nm laser set to a power density of 0.02 kW/cm^2^. In each well, 5 cells were imaged for 2000 frames at 50 ms exposure and 20 Hz.

The single-molecule images were analyzed using the Peak Fit plugin of the GDSC SMLM toolkit in Fiji [25]. Maxima were located using the difference of Gaussian filter. These were then fitted using a maximum-likelihood estimator and the precision was evaluated using the Cramer-Rao lower bound. Localizations with precisions better than 30 nm were then selected based on having a Gaussian width within 20% of the predicted PSF.

## 3. Results

### 3.1 Single-objective cantilever selective plane illumination microscopy

**Fig. 3:**
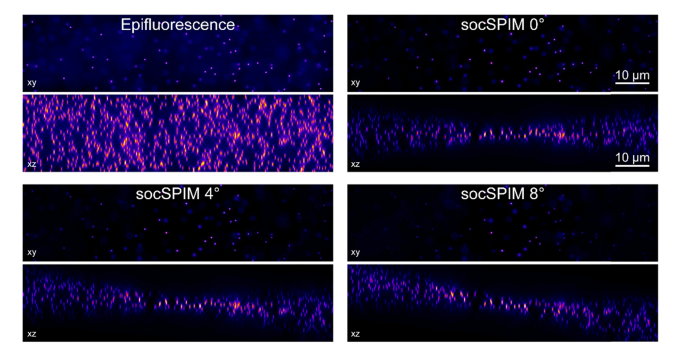
Simulations of how tilting the light sheet affects imaging. 1000 beads were randomly distributed in a 512×128×128 volume. The PSF of the beads was simulated using the PSF generator plugin [27] for Matlab. The xy data shows the beads as viewed by a 90× 1.27 NA objective lens, while the xz data shows a sum projection of all the beads as viewed from the side (without the objective lens). To generate the socSPIM data, the epifluorescence data was multiplied by the light-sheet intensity distribution as defined by the experimentally measured FWHM thickness and Rayleigh length.

**Fig. 4:**
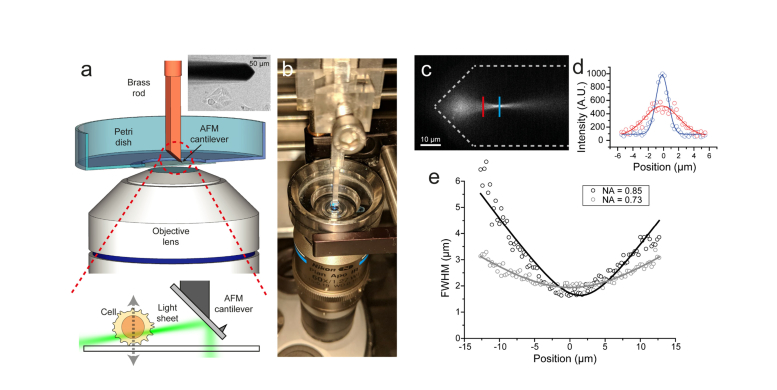
Principles of socSPIM. a: Schematic of the socSPIM setup. A reflective AFM cantilever is mounted onto a machined brass rod. This rod can be placed near to a high NA objective lens in a standard sample such as a petri dish with a coverslip bottom. The inset shows a white light image of the cantilever near HEK cells, with the reflective back surface of the cantilever facing the objective lens. A schematic of the socSPIM geometry is shown at the bottom, where 3D scanning can be achieved by moving the sample stage. The angled brass rod creates a slight downward tilt of the sheet to enable imaging near the coverslip surface. b: Photograph of the setup when imaging with a standard coverslip. c: Fluorescence image of Oxazine 725 excited by the light sheet setup with the cylindrical lens removed to determine the thickness of the sheet. The grey lines represent an approximate outline of the cantilever. d: Fluorescence profiles from (c) fitted with Gaussian distributions. e: Gaussian fits for the data in (d) were performed at each x-position for two different effective NAs, to evaluate the thickness as a function of axial distance and this was fitted to determine the Rayleigh length.

To overcome the typical requirements of custom-made sample chambers for LSFM, we developed an optical setup that introduces an external reflective surface to enable single-objective LSFM. An AFM cantilever was used due to its widespread availability and affordability. The AFM cantilever was attached to a machined brass or aluminium rod (Fig. 4(a)) that had an angled flat bottom, defining the angle at which the light sheet enters the sample. This value was chosen as 43° to provide a slight downward tilt of 4° (Fig. 4(a)), enabling imaging of the bottom surface of cells attached to the coverslip surface [14,26]. We simulated the effect of tilting the light sheet on acquired images (Fig. 3

) to ensure that tilting did not induce significant aberrations. The results show that the influence of tilting is minimal over the central 20x20 μm area, even for an 8° tilt, which is suitable for imaging cells.

While a machined rod simplifies the accurate attachment of the AFM cantilever, any angled surface could be used and in the first iteration we simply mounted the cantilever onto a countersunk bolt and attached it using cyanoacrylate glue. As our goal was to image in multiwell plates the machined brass rod was designed so that it and the cantilever would fit into the 5 mm diameter well of a 96-well plate. This small footprint also makes it compatible with most standard inverted microscope sample configurations, such as coverslips and petri dishes, with the only requirement being that the top surface of the sample is exposed. The cantilever can clearly be observed in white light transmission as seen in Fig. 4(a)

(inset), where the reflective back of the cantilever faces the objective lens to provide a mirror for LSFM. The flexibility of the cantilever allowed for trivial alignment a few microns above the surface, as it was possible to visually confirm when the cantilever came into contact with the surface by observing lateral motion. Despite deflecting upon physical collision with the glass surface, the flexible nature of the cantilever makes this design reasonably durable and in one case, a single tip was used for multiple experiments taking place over the course of one month without any noticeable degradation in the quality of the generated light sheet. The coating was also found to be compatible with μM concentrations of fluorophores without excessive adsorption in the presence of a blocking buffer. Naturally, with sufficient and repeated deformation of the cantilever the optical coating does scratch and delaminate, thus reducing the homogeneity of reflective surface and therefore the uniformity of the light sheet. At this point the cantilever can be replaced, using the same mounting surface after cleaning with a suitable solvent, such as acetone.

The cantilever light sheet setup was implemented on a commercial inverted microscope, which typically operates in TIRFM mode. Due to the longer path length of the excitation compared to the emission (Fig. 4(a)), the beam entering the objective lens was slightly decollimated using the beam expander that typically collimates the beam. This resulted in the divergent rays at the back focal plane producing a sheet of light in the imaging plane, albeit with a reduced effective NA. A cylindrical lens was used to focus one dimension of the laser beam to the back focal plane of the objective lens. While this arrangement is similar to other soSPIM implementations [11,16], due to the fact that the reflective surface is fixed in space, independently of the stage, it is not necessary to continuously modify the degree of decollimation, as samples are axially scanned (Fig. 4(a)). This enables facile 3D imaging through axial scanning of the sample stage or synchronous scanning of the objective lens and the cantilever. An adjustable circular aperture was used to generate a sheet with a suitable Rayleigh length [17] for imaging eukaryotic cells with typical sizes of 10-20 micron (Fig. 4(c)-(e)). We characterized the spatial distribution of the light sheet by imaging a fluorescent solution of 100 nM Oxazine 725 in PBS using 637 nm excitation. The axial thickness of the sheet could be determined by imaging the fluorescence without the cylindrical lens (Fig. 4(c)), as was done previously [11]. Using a 60× 1.27 NA water immersion objective, the light sheet had a thickness of 1.63 μm FWHM with a Rayleigh length of 4.3 μm (for an effective NA of 0.85) (Fig. 4(e)) and a beam propagation factor M^2^ of 2.20. By reducing the effective NA to 0.73 the Rayleigh length could be increased to 10 μm at the cost of an increased thickness of 1.94 μm (Fig. 4(e)) with an M^2^ of 1.34. It should be noted that the implementation of a bessel light sheet [28] or LITE microscopy [29] is compatible with the reflected light sheet and could therefore be used to maintain a thinner sheet over a larger range.

**Fig. 5:**
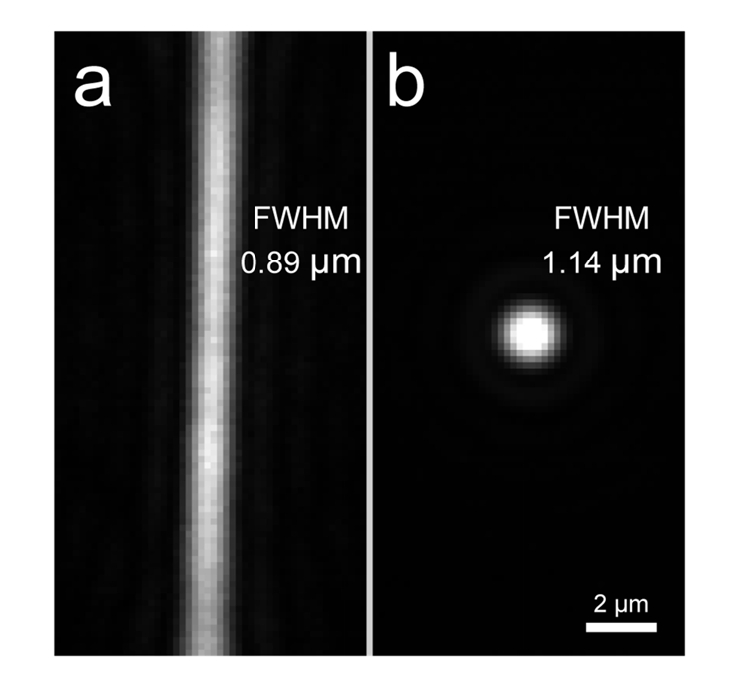
Imaging the light sheet using the reflection at the air-water interface. a: Dimensions of the light sheet in focus with the cylindrical lens. b: Same as (a) without the cylindrical lens.

We could confirm that the cylindrical lens did not greatly influence the thickness of the sheet by axially scanning the reflection created at the air-water interface when emission filters were removed (Fig. 5

). In most applications, we decided to use the 1.27 NA water immersion objective to limit spherical aberration, facilitate fast z-scanning and to maximize stability for repeatable super resolution imaging, but it was also possible to use a 1.49 NA oil immersion objective for enhanced single-molecule imaging.

**Fig. 6:**
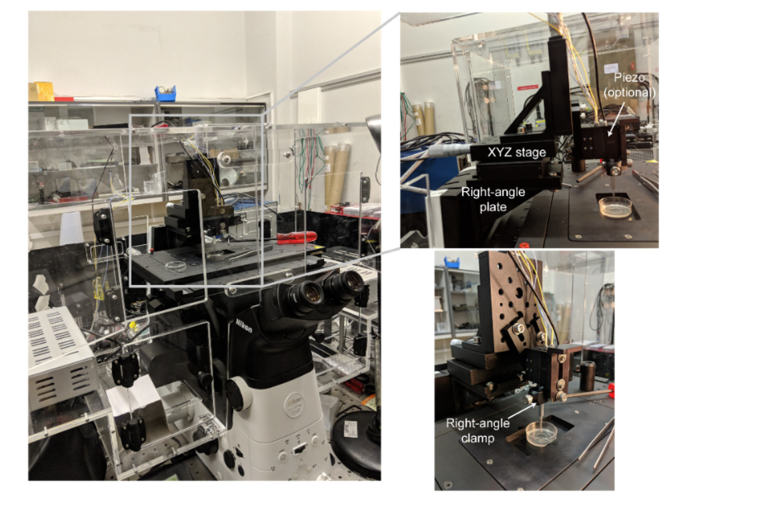
Simple implementation of the reflected light sheet. A second iteration of socSPIM was constructed on a standard inverted microscope with a typical microscope stage.

To demonstrate the flexibility and simplicity of this approach we implemented socSPIM on a second commercial microscope body (Fig. 6

) using standard parts that can typically be found in microscopy labs. We were able to efficiently recreate the setup using only a right-angle plate, an XYZ-stage, some brackets and a right-angle clamp (with an optional piezo-mount for automated scanning).

### 3.2 socSPIM imaging of the nuclear pore complex

We imaged the NPC in HEK cells labelled with Alexa Fluor 647-mAb414, to compare the performance of socSPIM with commonly used HILO excitation [4], and to demonstrate the ability of the new light sheet configuration to image in 3D. Scanning was performed by moving the stage axially, which maintains the relative position of the cantilever to the objective lens.

**Fig. 7:**
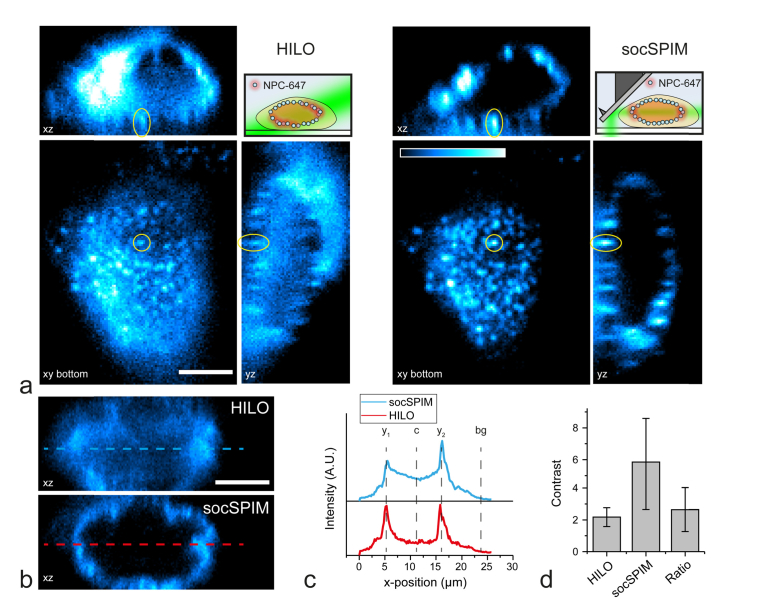
3D-imaging of the Alexa Fluor 647-labelled nuclear complexes in HEK cells. a: Orthogonal projections of 3D stacks of the same cell imaged using HILO and socSPIM. The xy view shows the bottom of the cell nucleus where NPCs can be easily identified (yellow circle). The xz and yz slices shown are centered on the highlighted NPC from the xy view. The cartoons depict the excitation modes. b: xz views of the same cells imaged using the two methods with red and blue lines indicating where intensity was analyzed for comparison. The scale bar is 5 μm. c: Average intensity plots taken over the lines in (b). d: Ratio calculated for n = 10 cells using intensity plots in (c). The error represents the standard deviation.

An orthogonal projection shows that the light sheet can be used to reconstruct clear images of the NPCs (Fig. 7(a)

). This result was directly compared to HILO excitation by imaging the same cells, showing that the use of LSFM is critical to reduce background fluorescence caused by out-of-focus excitation. NPCs could readily be identified, as indicated by the yellow circles in (Fig. 7(a)). By using a tilted sheet, it is possible to observe the same NPCs at the surface both using socSPIM and HILO, demonstrating that entire cells can be imaged using socSPIM. We imaged ten cells using both methods and compared the relative fluorescence at the membrane to the fluorescence at the center of the same cell in both imaging modes (Fig. 7(b)), which represents a contrast metric of the ability to limit out-of-focus excitation. The contrast was calculated using

$$\frac{\left(y_{1}-\text{bg}+y_{2}-\text{bg}\right)/2}{c-\text{bg}}$$

where y_1_ and y_2_ are the two peak intensities at the nuclear membrane, c is the minimum intensity at the center of the nucleus and bg is the background fluorescence outside of the cell (see Fig. 7(c)). This showed that under these conditions the light sheet enabled an approximately threefold improvement in relative contrast (Fig. 7(d)), commensurate with the expected difference in thickness of the sheet (~1.9 μm) compared to HILO (~6 μm [4]). These results suggest that the cantilever light sheet is suitable for 3D imaging and superior to standard HILO microscopy in terms of contrast.

### 3.3 Whole-cell 3D imaging of lysosomes

One of the advantages of LSFM, other than improving contrast, is fast 3D imaging with minimal photobleaching, particularly when compared to other excitation modes such as confocal microscopy. We demonstrate this ability by imaging HEK cells transfected with a plasmid coding mEos4b, genetically fused to the 5′-end of the rat LAMP1 gene in tandem, which codes for a transmembrane glycoprotein on the surface of lysosomes. Lysosomes are membrane-bound organelles responsible for the en bloc degradation of biological macromolecules and recycling of various cellular proteins [30]. They are highly dynamic structures that continuously change in size by undergoing fusion into larger or fission into smaller vesicles, which are better adapted to the physiological requirement [31].

**Fig. 8:**
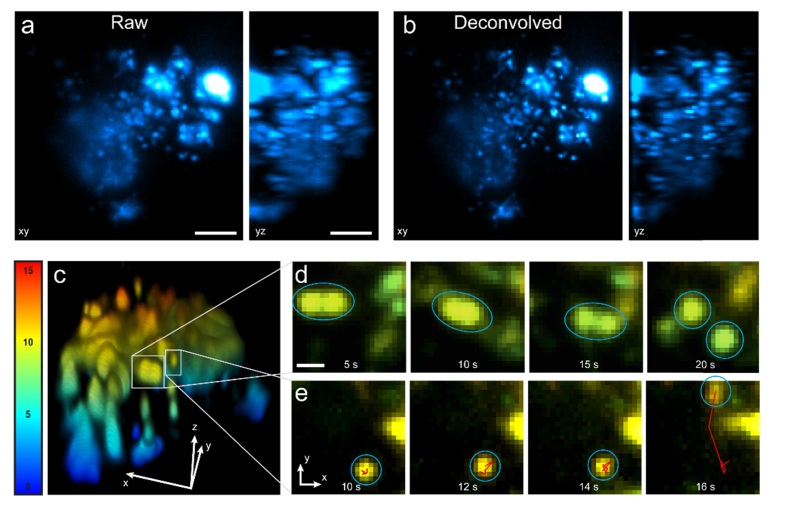
Live whole-cell 3D imaging using socSPIM. a: Maximum intensity projections in xy and yz at t = 10 s of lysosomes in HEK cells labelled with mEos4b-LAMP1. b: Same as (a) with Richardson-Lucy deconvolution. c: 3D volume rendering of deconvolved lysosome data, color-coded by depth in μm. d: A large complex diffuses slowly until it suddenly splits into two complexes as indicated by blue circles. e: An initially slowly diffusing lysosome can be seen to suddenly undergo fast, directed motion (red line shows the local 10 tracks backward). The scale bar in a-b is 5 μm and the scale bar in d-e is 1 μm.

Cells were exposed to a low amount of 405 nm excitation to activate a portion of the photoactivatable fluorescent protein mEos4b. The microscope stage was then continuously scanned using a piezo mount in steps of 500 nm, resulting in a whole-cell 3D volume frame rate of 0.5 Hz. Maximum projections of mEos4b-LAMP1 in a HEK cell are shown in Fig. 8(a)

. This data was deconvolved using the Richardson-Lucy algorithm to improve resolution (Fig. 8(b)). A 3D rendering of the deconvolved lysosome data is shown in Fig. 8c, color-coded by depth (also see Visualization 1). We could observe lysosomes undergoing fusion (Fig. 8(d)) and sudden directed motion (Fig. 8(e)), which are common characteristics of lysosome dynamics that demonstrate the ability of socSPIM to capture dynamics on the scale of a second with whole-cell 3D imaging.

### 3.4 Super-resolution imaging of the cell membrane using PAINT

**Fig. 9:**
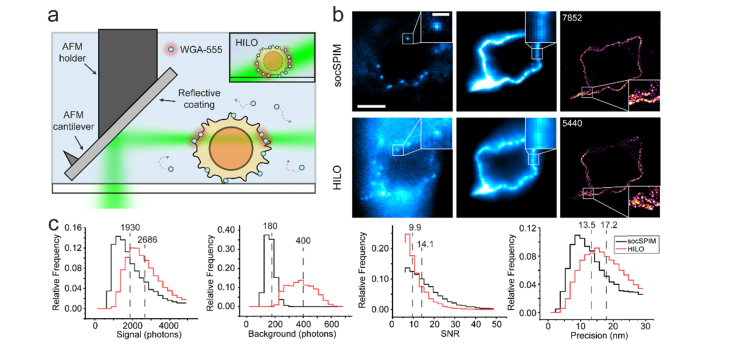
PAINT imaging using socSPIM. a: Schematic of PAINT experiment excited by a reflected light sheet. WGA labelled with Alexa Fluor-555 diffuses freely in solution until it encounters the cell membrane at which points it becomes immobilized. The inset shows HILO excitation, which excites a larger volume compared to the light sheet. b: The same cell imaged using PAINT with light sheet and HILO microscopy. The left image shows a typical single frame from a PAINT experiment. The middle image represents a diffraction-limited image taken prior to photobleaching of attached WGA. The right frame shows a reconstructed PAINT image using fitted localizations, the number of which is indicated. The scale bar in b is 5 μm (1 μm in inset). c: Various statistics from the experiment shown in b. The dotted lines show the median values.

As the cantilever soSPIM configuration enables the use of high NA objective lenses, it is suitable for single-molecule fluorescence imaging. To demonstrate this we performed super-resolution light-sheet PAINT imaging of the cell membrane (Fig. 9(a)

) of Jurkat T cells fixed in suspension immobilized on poly-L-lysine coated coverslips. Alexa Fluor-555 labelled wheat germ agglutinin (WGA), which binds to glycosylated proteins on the cell membrane, was added to cells at a concentration of 20 nM. The WGA protein in solution is sufficiently mobile to blur during the typical 50 ms exposure time, while protein that binds to the fixed cell membrane becomes static and appears as a single emitter that can be precisely localized.

We directly compared the performance of the light sheet in PAINT imaging mode with HILO excitation by imaging the same cell, to demonstrate the benefits of using LSFM for super-resolution imaging [10]. Compared to HILO, the light sheet greatly improved the contrast in single-molecule imaging, which could also be observed in the diffraction-limited images taken prior to performing the PAINT experiment (Fig. 9(b)). The excitation power densities were not perfectly matched in this experiment as HILO had a larger signal (median: 2686 photons) than socSPIM (median: 1930 photons) (Fig. 9(c) and Visualization 2). Despite this, socSPIM enabled an improvement in signal-to-noise ratio (SNR) (median: 14.1 vs 9.9), precision (median: 13.5 vs 17.2 nm) and localizations (7852 vs 5440) due to the significantly lower background (median: 180 vs 400 photons). This demonstrates that LSFM improves super-resolution imaging compared to standard HILO excitation.

### 3.5 Light-sheet super-resolution imaging in a 96-well plate

**Fig. 10:**
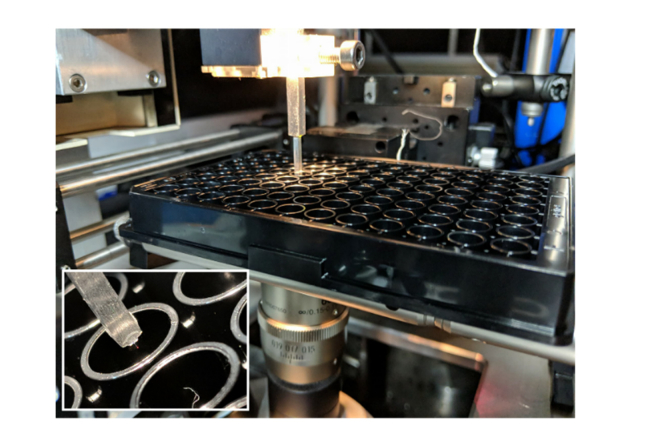
Setup used for LSFM in a 96-well plate. The inset shows the dimensions of the cantilever holder compared to the well and the AFM tip can be seen by the reflected red laser light.

The minimal footprint of the cantilever made it possible to use LSFM inside the wells of a 96-well plate (Fig. 10

and Fig. 11(a)

**Fig. 11:**
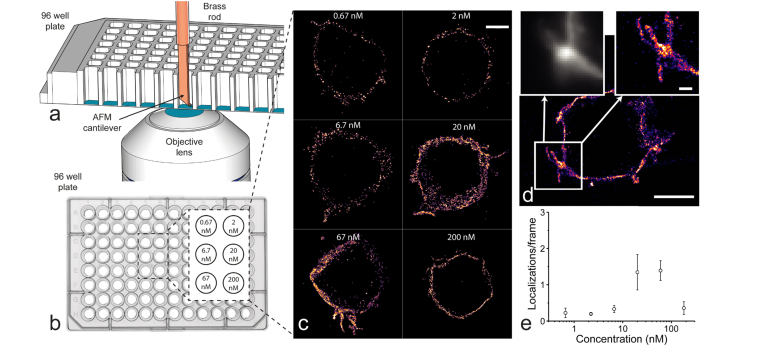
Super-resolution imaging of Jurkat T cells in a 96-well plate with socSPIM. a: Schematic demonstrating how the small-footprint cantilever mounted onto a rod fits into a 96-well plate. b: Schematic of a 96-well plate used in imaging experiments. The inset shows the six wells used with the varying concentrations of Alexa Fluor 555-labelled WGA. c: A sample super-resolution image taken over 4000 frames is shown for each concentration condition corresponding to (b). The scale bar is 5 μm. d: Image taken at the 67 nM concentration. The scale bar is 5 μm. The insets represent diffraction-limited (left) and super-resolution (right) magnifications of the region inside the white square. The inset scale bar is 1 μm. e: Variation of localization rate as a function of WGA concentration. The error bars represent the standard deviation in localization rate for n = 5 cells.

). We performed light-sheet super-resolution PAINT imaging of the cell membrane of fixed Jurkat T cells immobilized onto a 96-well plate coated with poly-D-lysine. Alexa Fluor 555-labelled WGA was placed in solution at concentrations ranging from 0.5 to 200 nM over six different wells (Fig. 11(b)).

**Fig. 12:**
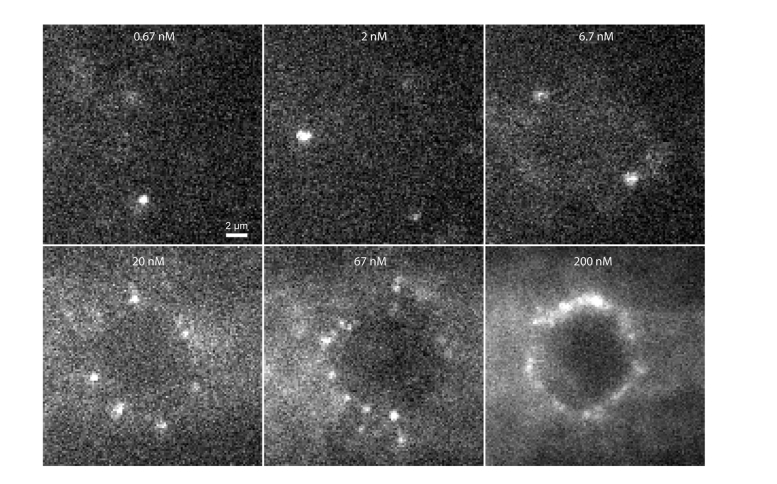
Sample frames used for constructing Fig. 11. The same contrast is used for concentrations 0.67 – 67 nM where single molecules can clearly be identified. At 200 nM the contrast had to be adjusted as it was too bright and at this point it became difficult to identify isolated emitters.

At low concentrations it would take a long time to accumulate sufficient points to create a super-resolution image, while at high concentrations there would either be too much background or too many emitters, preventing their localization. The results of increasing the concentration on imaging is shown in (Fig. 11(c)). 5 cells were imaged in each of the 6 wells over a 2.5 hour experiment under identical imaging conditions and with reconstructions performed using 2000 frames (4000 for image reconstructions in Fig. 11(c)). It was trivial to move between wells by translating the cantilever in z to clear the well and after changing wells approaching the sample again, where the cantilever would appear in the same position as before relative to the objective lens. No realignment was necessary when moving between wells. It can clearly be seen that increasing the concentration improves the resolution of the final image until the concentration becomes too large to image spatially isolated point spread functions of individual fluorophores. We quantified this observation by imaging 5 cells in each well and determining the localization rate (Fig. 11(e)). There is surprisingly little change over the lowest concentrations, perhaps suggesting that the localization rate there is limited by background fluorescence. As the concentration is further increased a sharp rise in localization rate can be observed, which then drops at the highest concentration, where it becomes difficult to distinguish individual emitters at 200 nM (Fig. 12

).

As WGA continuously binds to glycosylated proteins on the cell membrane it was possible to take a diffraction-limited light sheet image of the membrane prior to super-resolution imaging. Figure 11(d) shows how PAINT imaging enables the visualisation of small finger-like protrusions that are characteristic of T cells [32]. These are known to have diameters of 200-400 nm that would not be clearly discernible using diffraction-limited imaging. We could observe three distinct finger-like protrusions in Fig. 11(d) with FWHM diameters of 250 ± 50 nm that could not be resolved in the diffraction-limited image. These experiments demonstrate that socSPIM enables single-molecule and super-resolution LSFM in a 96-well plate.

## 4. Discussion

socSPIM offers a number of benefits compared to other LSFM implementations. Compared to oblique angle microscopy there is no loss in collection efficiency, however this comes with the added complexity of introducing an AFM tip into the sample. While lattice LSFM offers a thinner sheet and greatly improved resolution due to the use of bessel beams and structured illumination, this requires the use of dipping objectives, which limits its application when imaging standard samples. Furthermore, high NA oil objectives can be used with socSPIM and it should be possible to create a lattice light sheet using the reflective tip. In comparison to soSPIM, socSPIM enables z-scanning by only moving the stage similar to immersion versions of LSFM. Also, there is no need to tilt or refocus the light sheet as is the case with soSPIM. With our previous implementation of LSFM using custom-made sample chambers, we found that it could be particularly complicated to work with adherent cells that need to be grown in cell culture. socSPIM on the other hand is compatible with glass bottom petri dishes and multiwell plates, that are commonly used in biological and biochemical assays, which greatly simplifies the application of LSFM to standard inverted microscopes.

The most notable advantage of socSPIM is that there is currently no other way to perform high NA LSFM in multiwell plates. Automated super-resolution imaging of whole 96-well plates was recently demonstrated [9], which has great promises for high-throughput super-resolution imaging. This was done using TIRFM, but our implementation of LSFM enables high-throughput super-resolution imaging in multiwell plates with LSFM that drastically improves contrast, minimizes photobleaching and improves localization precision [10], when imaging above the coverslip. We demonstrated the feasibility of this approach here by imaging the membrane of Jurkat T cells using PAINT, showing that the light sheet improves contrast and precision compared to HILO. LSFM is particularly useful in PAINT applications [7] because the reduced thickness of the sheet compared to HILO allows the use of a higher concentration of probes, which increases the localization rate and allows for faster reconstruction of super-resolution images. This also applies to 3D PSF localization methods such as double-helix [33,34] and tetrapod [35], where the large increase in background fluorescence caused by the extended depth-of-field can be reduced [26]. When imaging using both 3D PSF localization and PAINT, optical sectioning would likely become a necessity.

The relatively simple and small footprint implementation of LSFM demonstrated here offers a low barrier of entry implementation of LSFM for users of standard inverted microscopes in terms of complexity and cost. Meanwhile, it also offers the ability to efficiently use LSFM with high NA objectives in standard samples, including multiwell plates, and socSPIM should therefore be useful to biologists and microscopists working on single-molecule imaging, super-resolution microscopy and high-throughput imaging.
